# Semi-empirical infrared spectra simulation of pyrene-like molecules insight for simple analysis of functionalization graphene quantum dots

**DOI:** 10.1038/s41598-023-29486-z

**Published:** 2023-02-09

**Authors:** Setianto Setianto, Camellia Panatarani, Deoraj Singh, I Made Joni

**Affiliations:** 1 Post Graduate School, Biotechnology Department, Padjadjaran University, Jl. Dipati Ukur No. 35, Bandung, Jawa Barat 40132 Indonesia; 2 Department of Physics, FMIPA, Padjadjaran University, Jl. Raya Bandung-Sumedang KM 21, Sumedang, Jawa Barat 45363 Indonesia; 3 Department of Biochemistry, Weill Cornell Medical College, New York City, NY USA; 4 Functional Nano Powder University Center of Excellence, Padjadjaran University, Jl. Raya Bandung-Sumedang KM 21, Jatinangor, 45363 Jawa Barat Indonesia

**Keywords:** Physics, Atomic and molecular physics, Chemical physics, Condensed-matter physics

## Abstract

The Infrared (IR) spectra usually assume the samples are 3D materials. Thus, it is difficult to identify functional groups in 2D materials at the edge and the center of the 2D surface. Therefore, it is crucial to introduce analysis methods that enable the investigation of 2D carbon materials such as graphene and its derivatives using IR spectra. This study calculates the infrared spectra of pyrene-like molecules as an insight for a simple analysis of graphene quantum dots using a semi-empirical method. These IR spectra were correlated to the electronic transition and charge distribution associated with functional groups. The IR spectra analysis focuses on comparing the pristine and functionalized molecule at the wavenumber 1400–2000 cm^−1^_,_ especially to identify the C=C stretching mode and 3000–3500 cm^−1^ for C–H and OH stretching. Moreover, the determination of excitation spectra was carried out to analyze the electronic transition of the molecules in the ultraviolet–visible region (200–900 nm) calculated using ZINDO method. The investigation of the pyrene-like GQD permitted the identification of the edge and center surface functionalization in 2D carbon materials.

## Introduction

Infrared spectroscopy (IR) is one of the most commonly used spectroscopic techniques to identify specific functional groups of complex materials. Absorbent groups in the infrared region absorb within a specific wavelength range. In this way, IR spectroscopy can be very sensitive for determining the functional groups of the sample, since different functional groups absorb specific IR radiation frequencies. The absorption peaks of molecules are obtained by comparing their measurement with spectral databases^1,2^. This can also be used for qualitative and quantitative analysis of complex mixtures of similar compounds. Molecular bonds are subject to different vibrations and rotations; consequently, atoms within molecules are unstable under external forces. However, the presence of functional groups in 2D materials at the edges and center of the 2D surface is quite difficult to identify since the IR spectra usually assume the materials are 3D. Therefore, it is crucial to introduce analytical methods that allow the investigation of 2D carbon materials, such as graphene and its derivatives using IR spectra.

Graphene quantum dots (GQD), a novel type of graphene nanomaterial, have attracted tremendous attention. GQD is nano-sized material that is of particular importance as functional material with a variety of applications across all disciplines. In order to examine the fluorescence of the synthesized GQD, optical spectroscopic studies are performed^3,4^, which reveal a red shift in the photoluminescence (PL) emission when GQD is functionalized with amine groups at different pH values^5^. The pH modification causes the protonation or deprotonation of the functional groups. The functional group also causes the structural deformation of the GQD in the aromatic ring. This deformation causes an energy level in the mid-gap that allows electron transitions to occur in this state^6^.

Generally, when the oxygen functional group of particles grows three-dimensionally it increases particle size. However, it is reported that the changes in the oxygen functional group of GQD as reaction times elapse revealed a blue shift in PL spectra means particle size decrease^7^. Thus, there is no guarantee that the particles are 3D, they may form 2D materials with unique functional groups either in the center or at the edge that causes different PL responses. In this condition, GQD may possess complex structures with unpredictable vibrational motions and interactions^8–15^. Many researchers employed FTIR to investigate functional groups in GQD; however, atomic or molecular dislocation is rarely observed since considered 3D material. For instance, the FTIR applies to identify sulfonic-GQD as rich in sulfonic and hydroxyl groups on their surfaces^9,16,17^. These functional groups improved the hydrophilicity and stability of the sulfonic-GQD in an aqueous system. Due to the dislocation of various active groups, it is difficult to correlate the informational FTIR spectra with their hydrophobicity-enhancing abilities. In contrast, with nitrogen-containing GQDs such as pyridine and nitrogen with low oxygen content, remarkable blue shifts^18^ in PL spectra were observed. The composition and structure of GQD are characterized by various spectroscopic techniques such as X-ray photoelectron spectroscopy (XPS), and FTIR. The infrared spectra of the prepared GQD showed absorption bands at 1289 cm^−1^ and 1223 cm^−1^, which are attributed to the C–O–C stretching vibrations^19,20^. However, the results did not indicate the position of the functional group attached to the GQD. Some researchers also introduced a simulation method to investigate the functional group of GQD on their corresponding FTIR. The infrared simulation of the GQD structures with edge oxygen and amino groups, was studied using the ab initio method^21^. As a result, the functional groups at the edge of GQD change the relative order of their energy levels, particularly the energy levels of the highest occupied molecular orbital (HOMO) and the lowest unoccupied molecular orbital (LUMO). FTIR spectral data from ab initio calculations can be used to determine the chemical composition of the functional groups in the GQD. Therefore, the position and location of these functional groups still need to be investigated to give corresponding different FTIR characteristics. Instead of using ab initio calculation, methods with simple and faster approaches are needed. We also see that mostly researchers use the DFT to study the vibrational mode of a molecule. However, the semi-empirical Austin Model1 (AM1) method is also capable and quite successful in simulating the vibrational spectrum of organic materials and agrees with the experimental results^22–24^. Therefore, AM1 consider the most helpful auxiliary tool for the FTIR spectroscopy identification of GQD.

In this study, we used a semi-empirical method to calculate the infrared spectra of pyrene-like molecules to provide insight for a simple analysis of functional GQD. The investigations explain the complex molecular level vibration of GQD correlated with their structure. These vibrations could also inform the sensitivity and selectivity of the molecule under investigation^25^. Therefore, this method may give an insight into the applications of FTIR spectra for 2D carbon materials analysis, especially pyrene-based molecules and other complex 2D materials. Geometry optimization and vibrational frequencies of the molecules (Benzene, Pyrene, and GQD) are determined and analyzed using the semi-empirical methods AM1. We analyzed the IR spectra of these molecules at the aromatics vibrations (1400–2000 cm^−1^), which is identified as C=C stretching mode. At the same time, the AM1 method successfully predicted infrared intensities and a spectral intensity pattern of GQD. We also reported the excitation spectra to review the electronic transition of the molecules in 200–900 nm by Zerner's Intermediate Neglect of Differential Overlap (ZINDO) method^26^. The excited spectra showed that the molecule under study was a GQD material.

## Molecular model and computational methods

### The initial molecular structure model.

**Figure 1:**
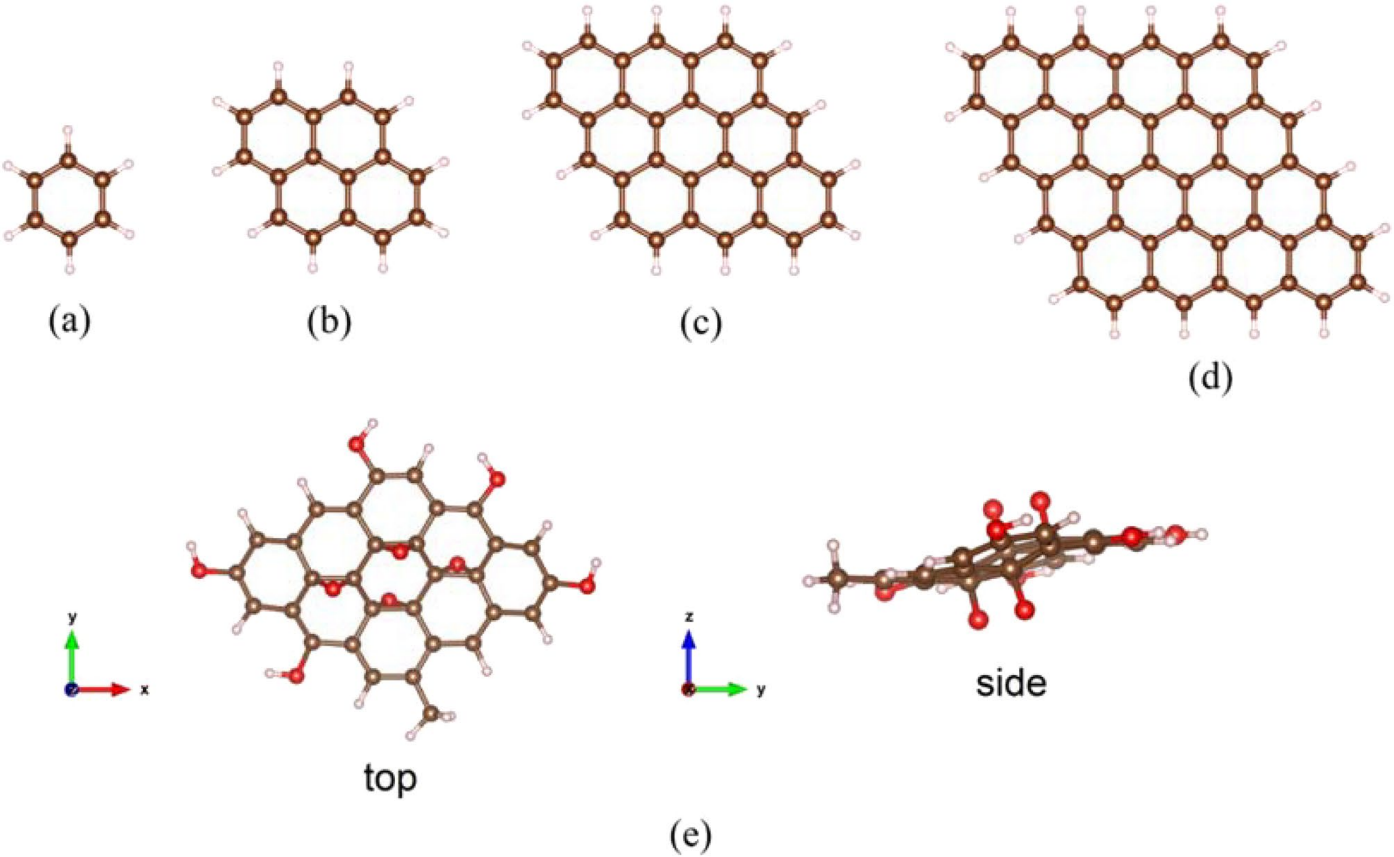
The initial molecular structure of Benzene (**a**), Pyrene (**b**), p1GQD (**c**), p2GQD (**d**), and fGQD with top and side views (**e**). Dark gold spheres represent carbon atoms, light white spheres hydrogen atoms, and red spheres oxygen atoms.

Benzene (C_6_H_6_) and Pyrene (C_16_H_10_) consist of their original condition of one and four-membered rings (Fig. 1a, b). Figure 1c and d show a nine and sixteen-ring pyrene extension model as pyrene-like GQD (pGQD). Throughout the manuscript, these pyrene-like GQDs with 9 and 16-ring structures are referred to as p1GQD and p2GQD. The model of the functionalized GQD is developed by adding five hydroxyl groups (–OH) and one methyl group (–CH_3_) on the edge and four oxygen atoms in the center of the surface of p1GQD, which are then termed functional GQDs (fGQD) as in Fig. 1e.

### Geometry optimization and IR simulation with the AM1 method

All calculations were performed on an Intel Core i5 computer with a 3.2 GHz CPU and 16.0 GB of physical memory. Optimizing the structures, vibrational modes, and excitation spectra were done using a Winmostar V10.0.7 package containing an AM1 semi-empirical method^27^. The initial geometrical structure of the pyrene molecule assumes to consist purely of regular networks with a CC distance of 1.397 Å, a CH distance of 1.084 Å, and bond angles of 120°. This approximation of the poly aromatic hydrocarbon (PAHs) refers to reported works^28^. Initially, all compounds were optimized using the molecular mechanic method. Then, an additional re-optimization by employing the AM1 semi-empirical method^29^. The convergence limit is determined based on orientation observations, i.e., after reaching the gradient limit of the energy change of 0.05 kcal/mol/Angstrom. The geometry optimization determines based on a second-order Taylor energy expansion around the current point^30^. Once a stable structure is available, the system provides the result of calculation data containing the energy and the electronic structure as an output file. A force calculation must then be carried out to obtain the vibration modes. The result matched against experimental data for that vibrational mode. The following calculation determines the absolute and percentage error in the predicted frequency. If the match between the calculated and the experimental frequencies is not close enough (within 10%), then the semi-empirical technique is not considered suitable for predicting that type of vibrational mode^31^.

### Excitation spectra determination according to the ZINDO method

The excitation spectra simulations for the structure of the geometry optimization results with AM1 were performed by one-step or single-point calculations using the semi-empirical ZINDO method^26^. The calculation consists of two parts. The first is the ground state calculation, which gives molecular orbital coefficients and eigenvalues. The ground state calculation is then followed by a configuration interaction calculation. The energy corresponding to a singlet–singlet transition between pure configurations is given by:

1 $$\Delta {E}_{ia}={\varepsilon }_{a}-{\varepsilon }_{i}-{J}_{ia}+2{K}_{ia}$$

where ${\varepsilon }_{a}$ and ${\varepsilon }_{i}$ are the orbital energies of orbitals a and i, respectively. ${J}_{ia}$ is the molecular Coulomb integral $(ii|aa)$ and ${K}_{ia}$ is the molecular exchange integral $(ia|ai).$ The calculated transition energies are then entered as diagonal elements of the Hamiltonian of the configurational interaction (CI). The off-diagonal elements of the CI Hamiltonian are:

2 $$\langle {}^{1}{\varphi }_{0}\left|H\right|{}^{1}{\varphi }_{i\to a}\rangle =0$$

3 $$\langle {}^{1}{\varphi }_{i\to a}\left|H\right|{}^{1}{\varphi }_{j\to b}\rangle = 2\left(ai|jb\right)-(ab|ij)$$

The calculation is done with CI restrictions and with a single excited CI, then the orbital calculation command is activated, and an electronic transition spectrum is generated in the form of a wavelength (λ) range and its oscillator strength. This determination of the excitation spectra was performed to examine the electronic transition level of pGQD in 200–900 nm. In addition, these data confirm that the studied compound is a quantum dot material.

### Analysis methods of vibrational mode and excitation spectra

#### Benzene

The infrared spectrum of benzene is characterized by several distinct peaks, which correspond to the vibrational modes of the molecule. In addition, the positions and intensities of these peaks are characteristic of the specific chemical bonds present in the molecule to identify the compound. The infrared spectrum of benzene helps determine the functional groups present in a compound and for structural analysis. Since the calculation results only provide line spectra, we used the Lorentzian function intended for comparison with the experiment. This spectrum simulates the broadening of the observed lines due to the finite width of the entrance slit and thermal motion^32^. In this case, we used the broadening function with a full width at half maximum **(**FWHM) of 40 cm^−1^. The vibrational frequencies generated by semi-empirical programs are often multiplied by a scaling factor to better match the experimental vibrational frequencies, i.e. 0.954 for the AM1 calculation^33^. Thus, simulations of the infrared spectrum can be used to analyze and provide important information about a molecule's structure and functional groups.

#### Pyrene, pGQD, and fGQD

The pyrene molecular mode vibrational pattern is used to refer to the original graphene quantum dot vibrational mode model (pyrene-like). By analyzing the intensity and pattern of the C=C (*sp*^2^) vibration at wavenumber (1400–1800) and (2900–3200) cm^−1^ for C–H (*sp*^3^) stretching on the pyrene molecule, they can be used to determine the microscopic properties of the pyrene-like GQD structure (Fig. 1c,d). It can be practically used in experiments to speed up the analysis of specific structures in pure GQD. Meanwhile, to analyze more complex functional GQD vibration patterns, we use the fGQD structural model as in Fig. 1e. Moreover, several different vibration patterns are used besides C=C (*sp*^2^) vibration and C–H (*sp*^3^) stretching, namely: C=O (*sp*^2^) vibration at 1600–1900 cm^−1^, OH stretching at 3000–3500 cm^−1^, and C–O bonds at 1000–1200 cm^−1^. The vibration pattern of the fGQD model can be used to analyze the results of IR spectrum measurements of GQD materials. The vibration patterns determine whether the molecule is pure GQD or functionalized GQD. The position of the functional groups, either at the edge or in the middle of the GQD surface, can also be qualitatively determined.

After analyzing the shape of the GQD molecular structure based on the vibrational mode pattern of the pyrene molecule, the next step is to investigate whether the excitation spectrum is in the UV (200–400 nm for pyrene) or in the visible (400–700 nm for pGQD) region. From the above two analyses, an electron distribution analysis using the molecular electrostatic potential (MESP) map was performed for the pristine and functionalized pyrene-like GQD models to know that they consequently affect the excitation. All of these analyzes were used to propose a mechanism, such as the electronic transition properties of the GQD pyrene-like model due to functionalization.

## Results

### Infrared spectra of benzene

**Figure 2:**
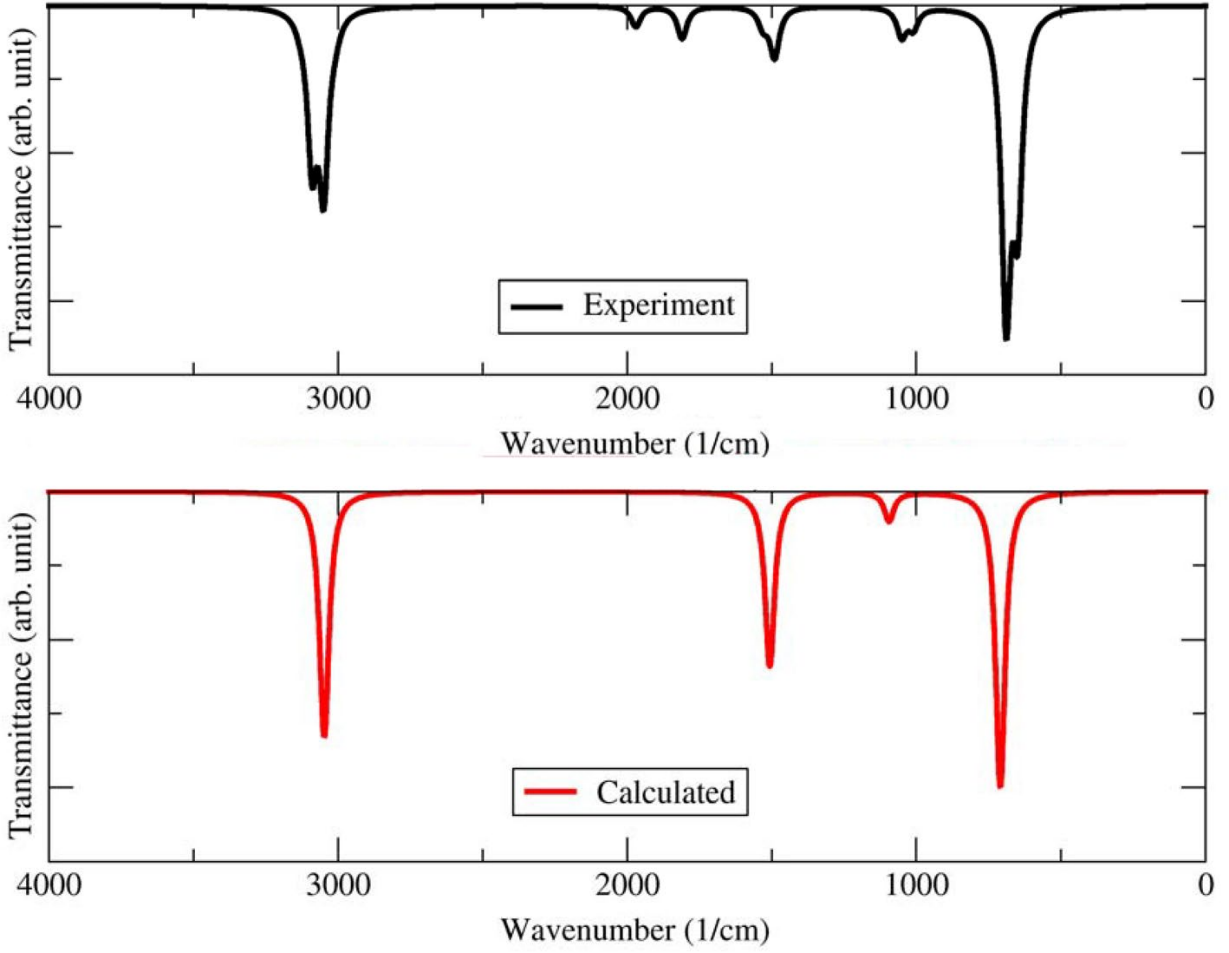
Infrared spectra of Benzene in the gas phase from experimental data (black) and calculation results (red).

The benzene's infrared (IR) spectrum is characterized by several absorption bands in the spectral region between 4000 and 600 cm^−1^. The most prominent absorption bands in the IR spectrum of benzene are due to the vibrations of the C–H bonds and the C=C double bonds in the molecule. However, there are also several other absorption bands in the IR spectrum of benzene due to the vibrations of other functional groups in the molecule. Figure 2 shows the calculation of the IR spectra of benzene in the gas phase. The vibrational motion of the benzene ring is not isolated but affects the entire molecule. The symmetric stretching and compression of all benzene carbons along the line is an example of ring breathing motion. The simultaneous expansion and compression of the six carbon atoms cause distinct circular breathing movements. The IR spectra of benzene showed four prominent peaks at 3047, 1506, 1093, and 713 cm^−1^ from the semi-empirical calculation (below) and 3062, 1486, 1038, and 673 cm^−1^ for the experimental measurement (above). This band represents both carbons bonded as vibrations of C–H and C=C (aromatic) groups. The peak at 3047 cm^−1^ wavenumbers represents the C–H stretch for the aromatic group. The peak at 1506 cm^−1^ is assigned to the C=C stretching frequencies of aromatic groups. The peak at 1093 is assigned to the in-plane C–H bend mode. The peak at 713 cm^−1^ wavenumbers represents the out-of-plane C–H deformation. In addition, weak peaks between 2000 and 1650 cm^−1^ in the experimental IR spectrum are a series of overtone and combination bands called the benzene fingers^34^. A detailed comparison of the vibrational modes of benzene molecules between experimental data and simulation results is given in Table 1.

**Table 1:** Mode Vibration of Benzene.

Vibrational mode (cm^−1^)^35^	Experiment^36^	DFT(*BP86/DEF2-SVP*)^37^ (error in percent)	AM1* (error in percent)
A2u C–H bend (1000–500)	673	663 ± 9.88 (1.49)	713 ± 42.35 (5.94)
E1u C–H bend (1500–1000)	1038	1030 ± 7.93 (0.77)	1093 ± 57.93 (5.30)
E1u C=C stretch (2000–1500)	1486	1465 ± 22.71 (1.55)	1506 ± 20.18 (1.34)
E1u C–H stretch (3150–2900)	3062	3114 ± 52.95 (1.70)	3047 ± 14.93 (0.49)

*This work, AM1 method with scaling frequencies 0.954.

Table 1 shows that the calculation results using the semi-empirical method agree with the experimental data for C=C and C–H stretching modes. The DFT and the semi-empirical method provide the same calculation results for the C=C stretching range with an error rate of less than 2%. The semi-empirical method agrees with experimental data with an error rate of less than 1% for the C–H stretch. Thus, infrared spectra simulations using the semi-empirical method give very acceptable results and agree with the experimental data for the range 3200–2900 cm^−1^ (C–H stretch) and 1800–1400 cm^−1^ (C=C stretch)^36^.

### The infrared spectra of pyrene and pGQD

**Figure 3:**
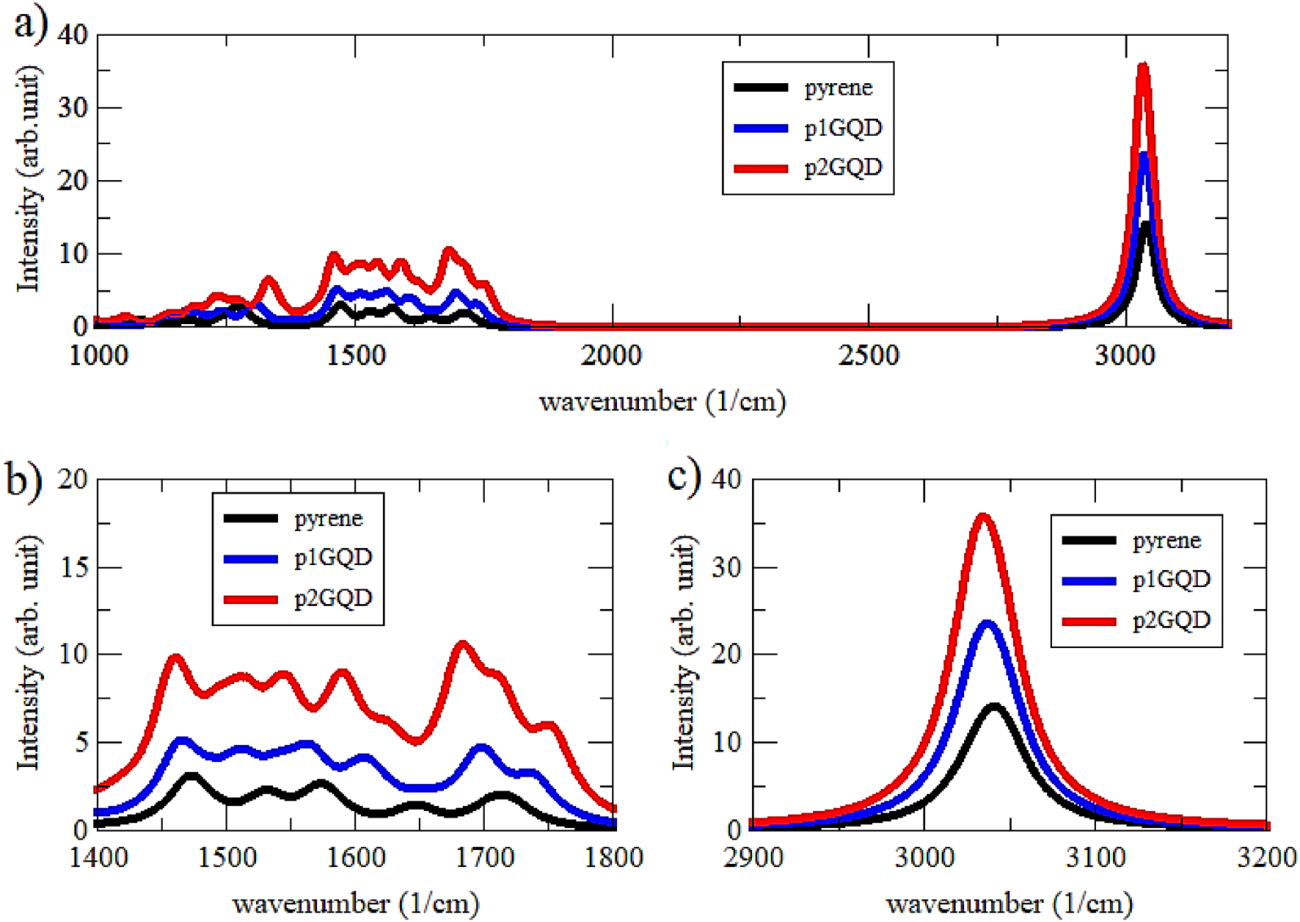
Simulation result of the vibrational mode (**a**), C=C stretch (**b**), and C–H stretch (**c**) of Pyrene, p1GQD, and p2GQD.

The infrared spectra of pyrene and pGQD are shown in Fig. 3 with two vibrational modes: 1400–1800 cm^−1^ for C=C aromatic stretch and 2900–3200 cm^−1^ for CH stretch^35^. The magnitudes and positions of these spectra are then compared to obtain a qualitative theoretical prediction of the molecular structure of GQD. The properties of the aromatic CC and CH stretching regions are first considered to understand the molecular structure of GQD. This stretch is characteristic of aromatic vibrations in 1400–1800 cm^−1^ (Fig. 3b) associated C=C stretch of pyrene, p1GQD, and p2GQD molecules.

The intensity increases as the size of the molecules increases. This increasing intensity is due to the fact that the number of carbon atoms is increasing. Therefore, we hypothesize that the properties of pGQD molecules can be identified using the IR spectra over these regions. Figure 3a is a plot of the full spectrum simulation results for the vibrational modes of pyrene, p1GQD, and p2GQD in the wavenumber range of 1000–3200 cm^−1^. At the same time, the simulation result of the CH stretching vibration of pyrene, p1GQD, and p2GQD is shown in Fig. 3b. It shows that the intensity increases sharply above 2900–3200 cm^−1^. This increasing intensity arises from the increasing number of CH bonds at the edge of the aromatic compounds. In detail, the vibration mode is given in Table 2. From the three analyzes of the vibrational modes, a qualitative analysis of the GQD molecular structure can be performed by examining the character of the infrared spectrum pattern in the region of the aromatic C=C stretching vibration around 1400–1800 cm^−1^. This pattern does not change significantly. However, the influence of the size of the GQD molecule can be quickly recognized by the increasing intensity in this area.

**Table 2:** The vibration mode of Pyrene, p1GQD, and p2GQD.

System	C–H stretching		C=C stretching	
	Wavenumber (cm^−1^)	Intensity (arb. unit)	Wavenumber (cm^−1^)	Intensity (arb. unit)
pyrene	3041	14.03	1712	1.99
			1648	1.43
			1574	2.63
			1532	2.25
			1473	3.10
p_1_GQD	3037	23.50	1699	4.70
			1607	4.11
			1563	4.94
			1513	4.63
			1466	5.13
p_2_GQD	3035	35.70	1752	6.00
			1685	10.60
			1591	9.01
			1545	8.90
			1512	8.73
			1462	9.80

### Excitation spectra of pGQD

The physical properties of semiconductor nanocrystallites are dominated by the spatial confinement of excitations (electronic and vibrational). Quantum confinement, the widening gap between the highest occupied molecular orbital (HOMO) and the lowest unoccupied molecular orbital (LUMO) with decreasing crystallite size, and its implications for the electronic structure and photophysics of the crystallites have attracted considerable interest^38^. In pi systems, the HOMO–LUMO Energy gap is so small that absorption occurs in the visible rather than the UV region of the electromagnetic spectra. If interaction with infrared light causes vibrational transitions in molecules, the interaction of light with shorter wavelengths in the UV (200–400 nm) and visible (400–700 nm) electromagnetic spectrum causes organic molecules to undergo electronic transitions. When energy from UV or visible light is absorbed by a molecule, one of its electrons is excited from lower energy into a higher energy molecular orbital.

**Figure 4:**
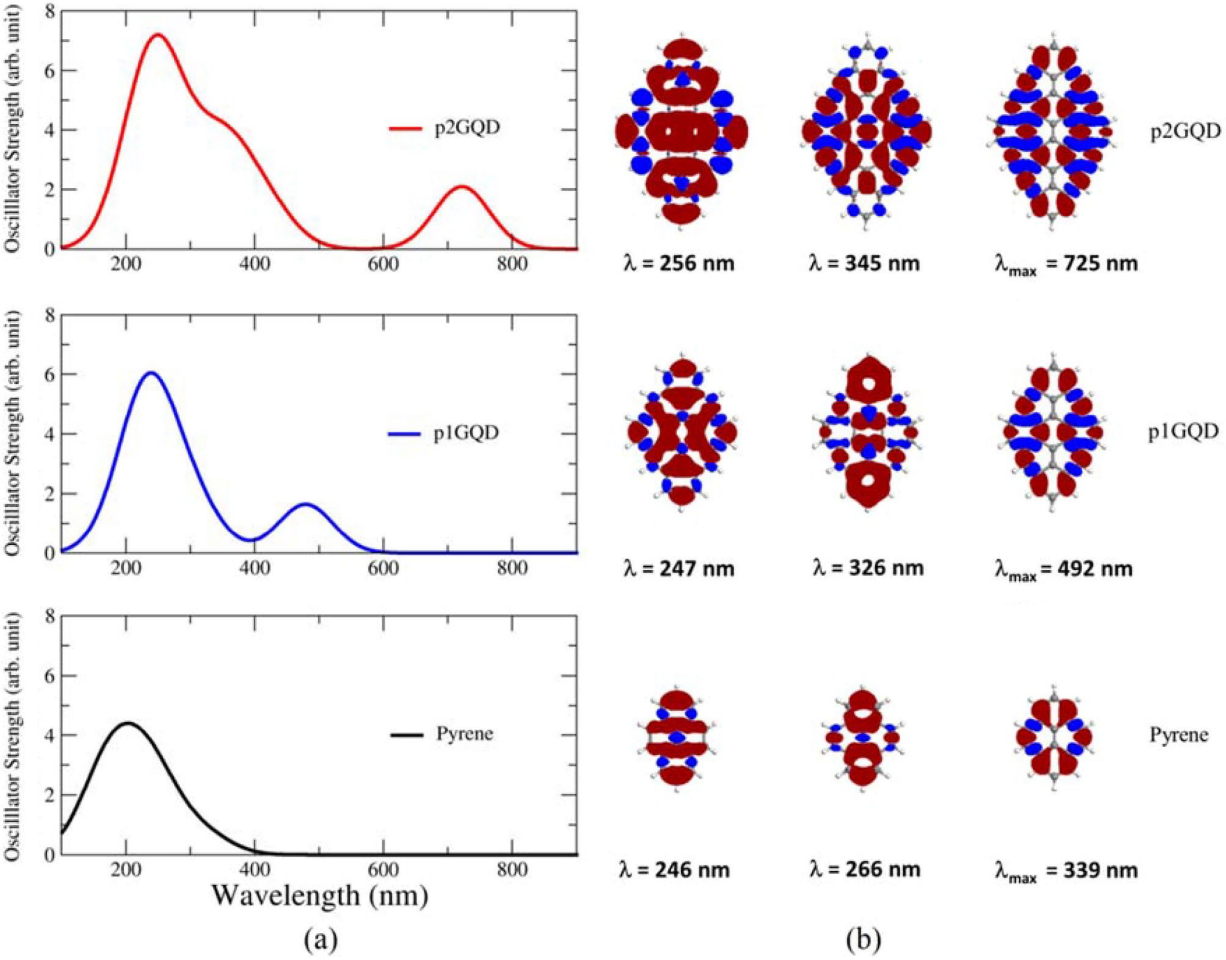
(**a**) Simulation result Excitation spectra of Pyrene (black), p1GQD (blue), and p2GQD (red) and (**b**).Charge difference density (CDD) map for the absorption peaks of Pyrene, p1GQD, and p2GQD. The blue color represents positive holes, and the red represents negative electrons.

The absorption spectra of pyrene, p1GQD, and p2GQD molecules with diameters of 9.23, 13.47, and 17.73 angstroms are shown in Fig. 4a. From the three excitation curves, the spectra clearly show the quantum confinement effect. The absorption of pyrene, p1GQD, and p2GQD shifted dramatically from the wavelengths of 246, 492, and 725 nm, respectively. This shows that the absorption spectra of GQD molecules with sizes ranging from 0.9 to 1.8 nm in diameter towards the red wavelength are shifted (redshift). This indicates that the pGQD molecule has the character of a quantum dot material^39^.

Additionally, a charge difference density (CDD) map of pyrene, p1GQD, and p2GQD at specific wavelengths is shown in Fig. 4b. The CDD map is a graphical representation of the difference in electron density between the ground state and the excited state, which can reveal changes in the electron distribution in the excited state. CDD maps of the absorption peaks for pyrene (λ_max_ = 339 nm), p1GQD (λ_max_ = 492 nm), and p2GQD (λ_max_ = 725 nm) show a similar pattern of alternating positive and negative charge densities along with conjugated C–C bonds. This character comes from the HOMO to LUMO (π → π*) transition in the system^40^. Meanwhile, the CDD map for absorption peaks at wavelengths other than at λ_max_ is a feature associated with the π → π* transition from the orbital below HOMO to above LUMO.

The p1GQD exhibited absorption in the UV range (100–350 nm) and in the visible range (400–600 nm) as shown in Fig. 4a. Thus, p1GQD was then used as a model for functionalized GQD (fGQD) by considering on several studies stating that the absorption peak of quantum dot material is in the visible range^41–43^.

### Infrared spectra of fGQD

**Figure 5:**
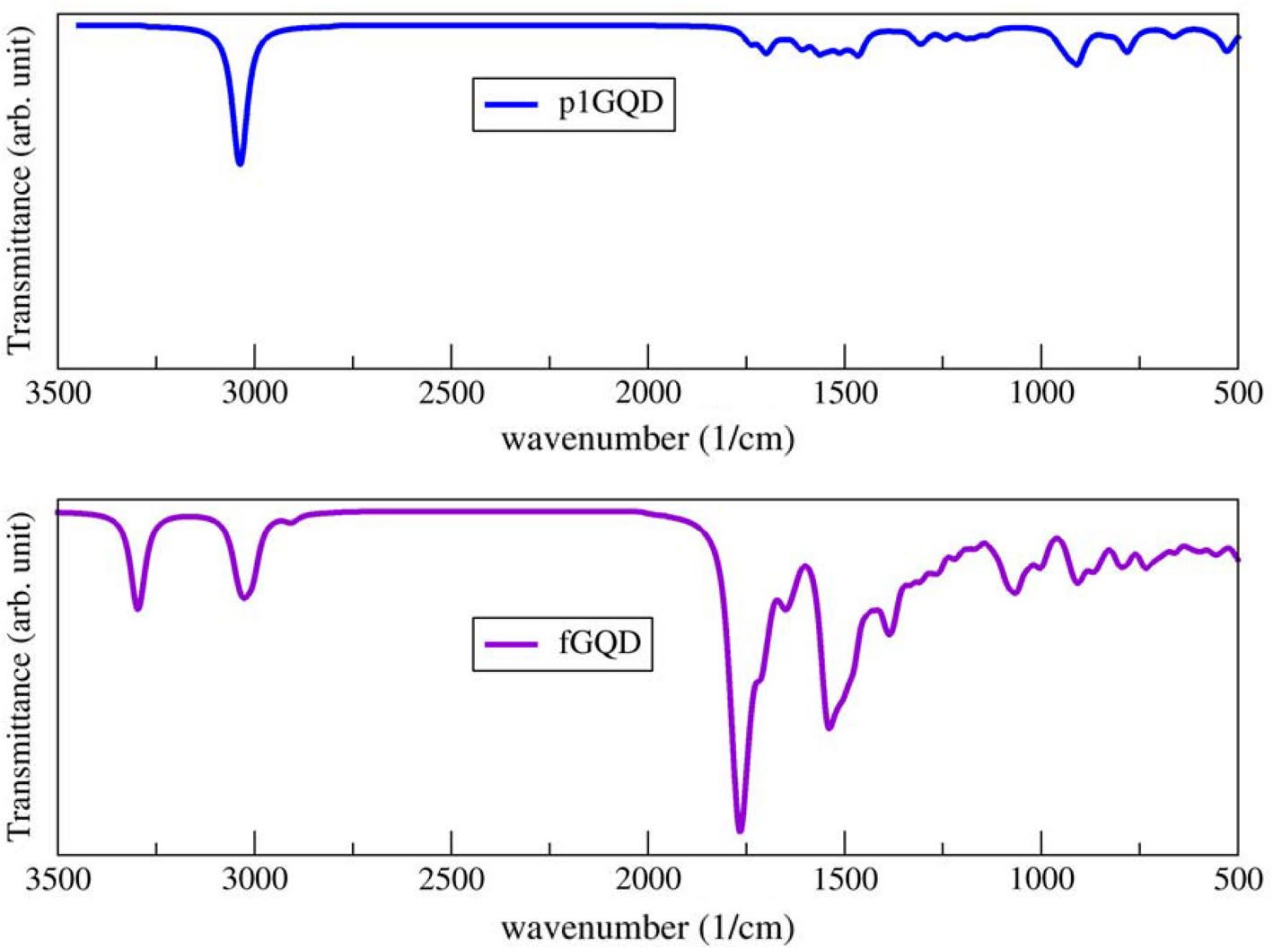
Simulation result of infrared spectra of p1GQD (blue) and functionalized fGQD (purple).

Infrared spectra for the pristine p1GQD compared to the functionalized fGQD (with adding a hydroxyl group (–OH) and a methyl group (–CH_3_) at the edges and four oxygen (O) atoms in the middle of the surface) are shown in Fig. 5. The spectra of fGQD differ from those of pure p1GQD. Comparing the fGQD with the p1GQD clearly showed that most of the rich chemical groups appeared in fGQD. The peaks in the regions 3300–3200 cm^−1^ and 3000–2900 cm^−1^ in fGQD spectra are assigned to OH stretching and CH (*sp*^3^) stretching. These peaks disappeared completely in pristine p1GQD, indicating that the edge of fGQD is functionalized by methyl and hydroxyl groups. The strong peaks at about 1786, 1650, and 1521 cm^−1^ are associated with the vibrations of C=C (*sp*^*2*^), C=O (*sp*^2^), and COH bonds, respectively, and a peak at 1059 cm^−1^ is associated with CO alkoxy groups on fGQD. These data reflected that fGQD had many oxygen-containing functional groups on their surfaces. All of these vibrational bands had a similar trend as the grapheme-dots (G-dots) sample observed at 3425, 1645, and 1078 cm^−1^ and associated with OH, CO (*sp*^2^), and CO (alkoxy groups) vibrations, respectively^44^. It is emphasized that our proposed method is capable of presenting unique splitting spectra in response to the edge functionalization and also a remarkable increase of the peaks in response to the center functionalization.

### Molecular electrostatic potential (MESP) maps of fGQD

**Figure 6:**
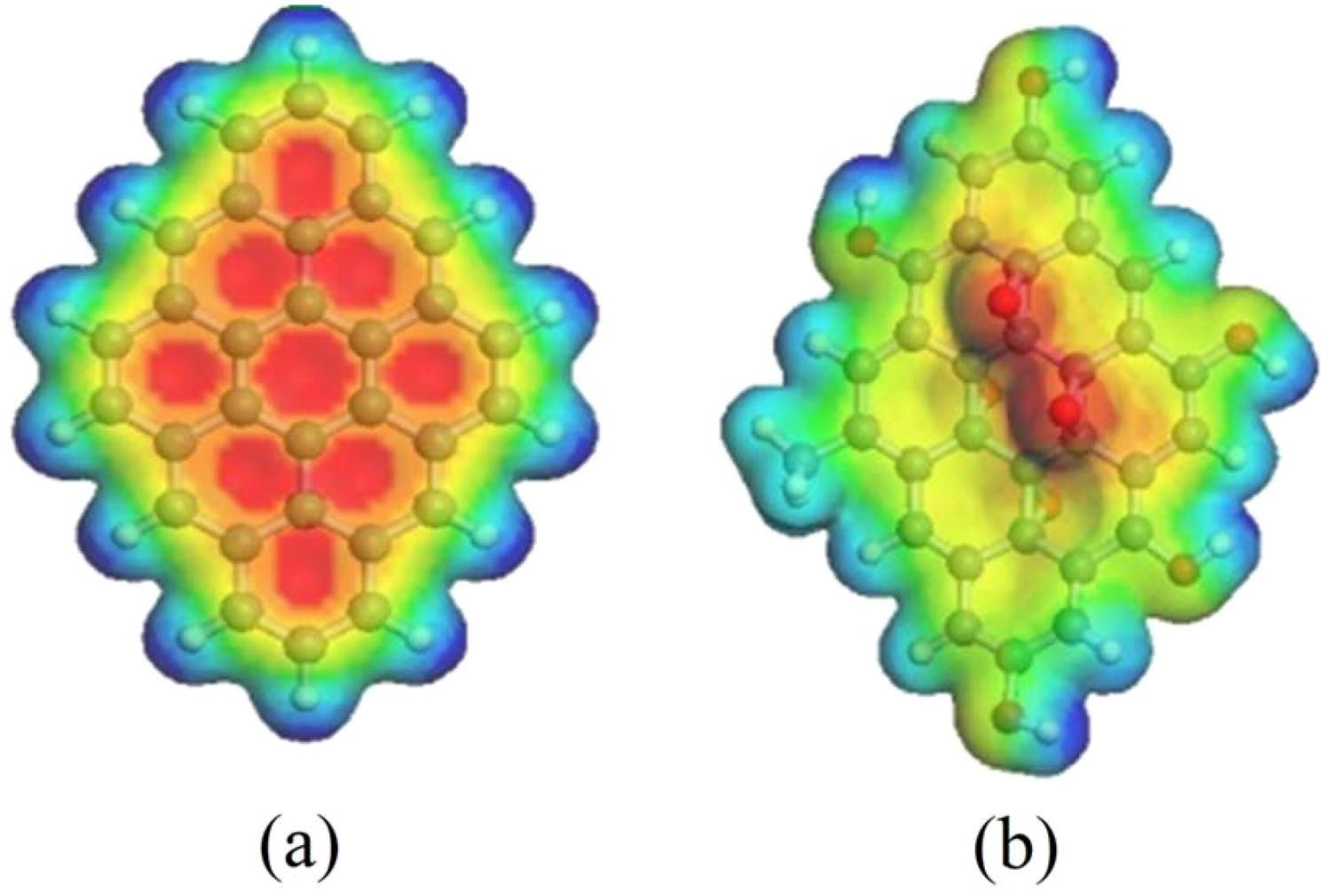
Molecular electrostatic potential (MESP) map of p1GQD (a) and fGQD (b). Electrostatics potentials are mapped on the surface of the electron density with the isosurface value of 0.1 unit. The red surface corresponds to a region of negative electrostatic potential, whereas the blue color corresponds to the positive potential.

Molecular electrostatic potential (MESP) maps are generated for the investigated chemical structures using the semi-empirical AM1 method. As a rule, they offer a simple and thoroughly positive way of explaining the charge distribution over a substance. Figure 6 lists the constructed MESP maps for pristine and functionalized pyrene-like graphene quantum dots (p1GQD and fGQD). The maps were created to consist of multiple colors ranging from red to dark blue. These colors appear in the order of red, orange, yellow, green, light blue, and dark blue from most negative to most positive regions, with red representing the extremely negative potentials and dark blue representing the positive ones. Also, yellow areas have fewer negative potentials than red areas, and green areas reflect areas with neutral potentials. Due to the electronegativity of the attached atoms, the potential distributions and colors can also be correlated to a certain extent. Atoms with high electronegativity appear red when connected to another less electronegative one. Hence, we can use the MESP maps as a physical property to decide whether the active sites of interest can undergo either nucleophilic or electrophilic chemical interactions ^45^. Regarding the MESP map of p1GQDs, three main colors make up the constructed map; red, blue, and dark blue. It is noteworthy that red is concentrated in the centers of benzene rings, triggering the effect of the electron delocalization phenomenon within the ring centers, suggesting that p1GQD is most likely to undergo nucleophilic interactions. While the terminals are characterized by light and dark blue colors, this can be attributed to the presence of less electronegative H atoms. Electrophilic reactions are most likely to occur here. For fGQD with four oxygen atoms at the center of the surface, the red color around the oxygen atom indicates a strongly electronegative region. This reflects the success of the electronegativity of oxygen in altering the distribution of delocalized electrons on the p1GQD sheet. Therefore, the electron distribution in the fGQD surface is indicated by a generally yellow color, except for certain red areas on the top surface where O atoms are present. This suggests that adding oxygen leads to a less negative structure of fGQD.

### Molecular electronic transitions of fGQD

**Figure 7:**
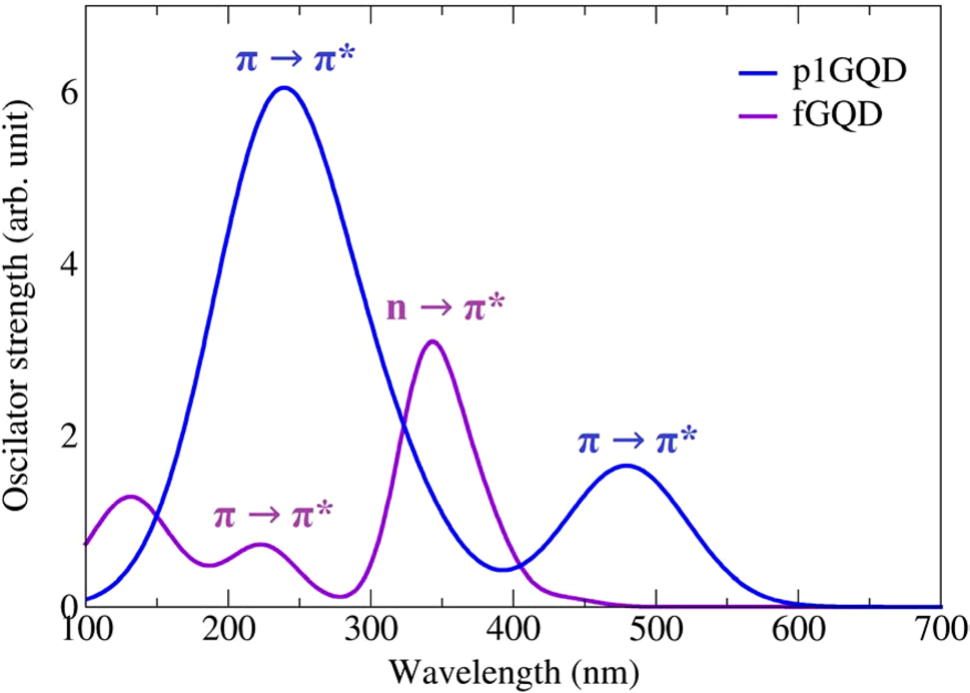
Simulation result of the excitation spectra of p1GQD (blue line) and fGQD (purple line).

Electron transitions in molecules occur when electrons in a molecule are excited from one energy level to a higher energy level. This process is denoted as a σ → σ*, π → π*, and n → σ* transition^46^. The energy changes in this transition event can provide information about the molecular structure and determine the molecular properties that are generally related to color^47^. The electronic transitions in p1GQD and fGQD molecules are given in Fig. 7. fGQD has a characteristic absorption band below 300 nm due to the weak π → π* transitions of aromatic C=C bonds, and broadband between 350 and 450 nm (or a shoulder at ~ 370 nm) due to strong n → π* transitions of C–O–C bonds. In p1GQD, the peaks of the π → π* transition are located at 247 and 492 nm. These results indicate that there is a change in the absorption spectra from λ_max_ = 492 nm (p1GQD) to λ_max_ = 370 nm (fGQD) due to the presence of C–O–C bonds in the middle of the p1GQD surface.

### Mechanism electronic transition of functionalized pyrene-like GQD

An electronic transition mechanism from the functionalized GQD is presented below using many aspects from the molecular structure, IR spectra, MESP map, and excitation spectra given in Table 3. First, we analyzed the intensity and the pattern of the C=C (*sp*^2^) vibration of the pyrene molecule at wavenumber 1400–1800 cm^−1^ and its excitation spectra in the UV range (200–400 nm), as well as the MESP map of the pyrene molecule, whose electron distribution belongs to the benzene ring, is delocalized. As the molecular size increases in p1GQD (9 rings) and p2GQD (16 rings), the intensity of the vibrational mode C=C (*sp*^2^) increases only slightly (weak to medium vibration), but the electron distribution pattern remains delocalized in the center of the ring, but the size effect affects the excitation spectrum, which shifts from the UV to the red wavelength (red shift), indicating that the molecule is a pyrene-like graphene quantum dot (pGQD). If the intensity and pattern of vibration mode C=C (*sp*^2^) has increased drastically (moderate to strong vibration) and another vibration mode appears, i.e. O–H stretch (3000–3500 cm^−1^), the GQD molecule is fGQD pyrene-like. The electron distribution on the surface is disturbed and causes a shift in the excitation spectra towards the blue/violet wavelength (blue/violet shift). This transition occurs due to the transfer of electrons from a nonbonding pair of electrons to an antibonding orbital (n → π*) which is caused by the presence of the lone pairs for four oxygen atoms in the center of the surface of p1GQD.

**Table 3:**
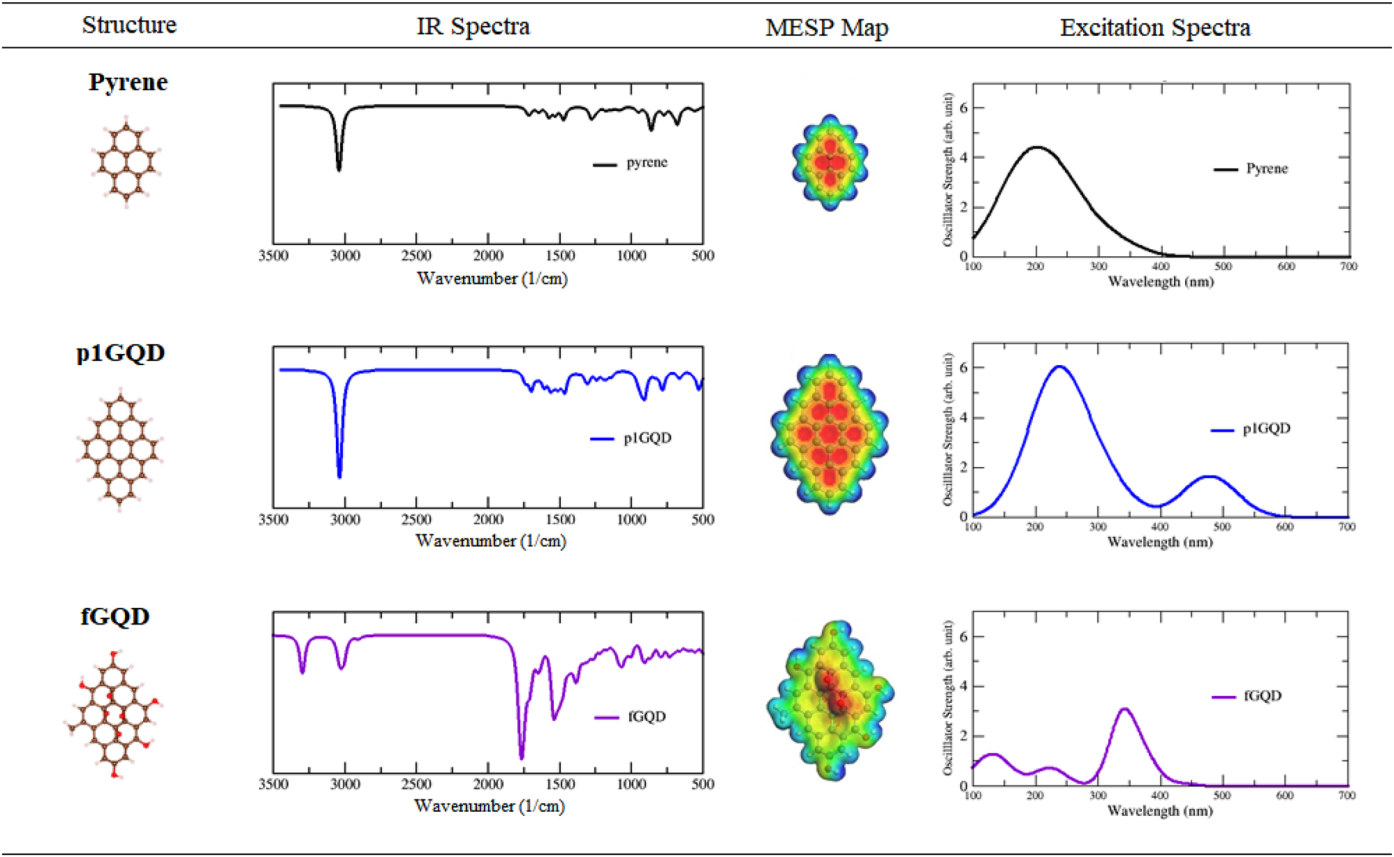
Various pyrene-like molecular structures and their corresponding IR, MESP map, and excitation spectra.

## Conclusion

The proposed model of IR spectral analysis using semi-empirical methods successfully identified the presence of peaks corresponding to the edge and center functionalization of pyrene-like GQD and can be applied to 2D materials in general. The edge functionalization of GQD is characterized by the presence of two unique peaks in the vibration spectrum at wavenumber 3300–3200 cm^−1^ and 3000–2900 cm^−1^ while, the presence of functional groups at the center of the GQD surface is indicated by strong peaks in the vibration spectrum at wavenumbers about 1786, 1650, and 1521 cm^−1^. It was also found that due to the presence of functionalizing groups, the charge distribution was localized on the oxygen atoms, resulting in a blue shift in their excitation. Therefore, we hypothesize that the transition occurs due to the transfer of electrons from a nonbonding electron pair to an antibonding orbital (n → π*).

## Data Availability

All data used for this study are contained in this article.
